# Neurological Complications Secondary to Intimate Partner Violence: A Brief Review and Case of Posterior Cerebral Artery Cerebrovascular Accident Following Domestic Abuse

**DOI:** 10.7759/cureus.42823

**Published:** 2023-08-01

**Authors:** Alexandra C Skoczek, Akram Haggag

**Affiliations:** 1 Medicine, Edward Via College of Osteopathic Medicine - Auburn, Huntsville, USA; 2 Internal Medicine, Crestwood Medical Center, Huntsville, USA

**Keywords:** clinical case report, abusive traumatic brain injury, neurology, magnetic resonance angiography (mra), cerebrovascular accident (stroke), domestic abuse, intimate partner violence (ipv)

## Abstract

Intimate partner violence (IPV) is a growing public health concern, with millions of individuals experiencing IPV each year. Consequences of IPV include psychological disturbances, changes in physical health, and in extreme cases, severe disablement or death. Here, we describe a case of a patient who experienced IPV, leading to a variety of neurological symptoms, and was diagnosed with a posterior cerebral artery (PCA) cerebrovascular accident (CVA) 10 days later. While cases of traumatic brain injury leading to CVA, or stroke, have been documented, there is currently limited reported literature on the neurological complications, specifically stroke, secondary to IPV in adults. Due to this limited reporting, future studies on IPV will be needed to fully understand the long-term neurological complications that may occur.

## Introduction

Intimate partner violence (IPV) is defined as any physical or sexual violence, stalking, and/or psychological aggression by a current or former dating partner or spouse [1]. An intimate partner is any person, regardless of gender, with whom an individual has a close personal relationship. This close personal relationship can include emotional connections, physical contact, and/or sexual behavior. Intimate partners do not need to identify as a couple, may or may not be cohabiting, and can be of the opposite or same sex [1]. Physical violence is defined as a physical force that has the potential to cause death, disability, injury, or harm and includes, but is not limited to, pushing, shoving, throwing, choking, shaking, punching, hitting, burning, or use of a weapon [1]. In 2015, The Centers for Disease Control and Prevention updated the definitions of IPV, intimate partners, and physical violence, as IPV has become a growing national public health concern [1,2].

An estimated 59 million women and 52.1 million men in the United States have experienced IPV in their lifetime [2]. Victims of IPV experience a variety of acute and chronic medical conditions both emotional and physical. The World Health Organization (WHO) Multi-Country Study on Women's Health and Domestic Violence found that female victims of IPV have increased odds of depression, post-traumatic stress disorder (PTSD), anxiety disorders, sleep difficulties, eating disorders, and suicide attempts [3,4]. In addition to psychological consequences, female victims also self-report poor or very poor general health and a variety of different neurological symptoms such as difficulty walking, dizziness, headaches, and memory loss [5,6]. In the following case, we discuss a unique presentation of a woman who was a victim of IPV and subsequently experienced a cerebrovascular accident and experienced a variety of neurological symptoms including vision loss and headaches. 

## Case presentation

A 45-year-old female with a past medical history of hypertension presented to the emergency department (ED) with vision changes for the last 10 days. The patient reported that she was assaulted by her partner 10 days prior and had been struck in the front of her head with a closed fist. She denied losing consciousness at the time but said that since the incident she had been experiencing vision changes, headaches as well as subjective dizziness, left-sided weakness, and left-sided numbness. She described the vision changes as a blurry loss of vision in her left eye that had been constant as well as a constant headache. The weakness and numbness had been improving but were still minimally present. The patient noted that she normally wears glasses at home but said that her vision was not improved with her glasses.

The patient stated that after the incident 10 days ago, she had presented to a different ED for her symptoms and underwent a head CT. However, she left the ED before receiving the results. Physicians at that ED had told her she most likely had a concussion and that her symptoms should resolve. While her weakness and numbness had been improving her continued vision loss and headaches prompted her to return to the ED. On this ED visit 10 days later, she denied speech difficulties, difficulties swallowing, or any other symptoms. On neurological exam, the patient had a visual acuity of 70/20 in the left eye and 50/20 in the right eye. Baseline visual acuity was unknown, but the patient subjectively reported that this was “much worse” than her baseline. Pupils were equal, round, and reactive to light with extraocular motion intact. Cranial nerves II-XII were intact with no focal motor or sensory deficits appreciated on examination. Cerebellar testing including Romberg’s test, heel-to-shin, and rapid alternating movements was normal. The station and gait were intact, and the patient was alert and orientated x3. The rest of her examination was unremarkable. Vital signs and labs were all within normal limits.

**Figure 1:**
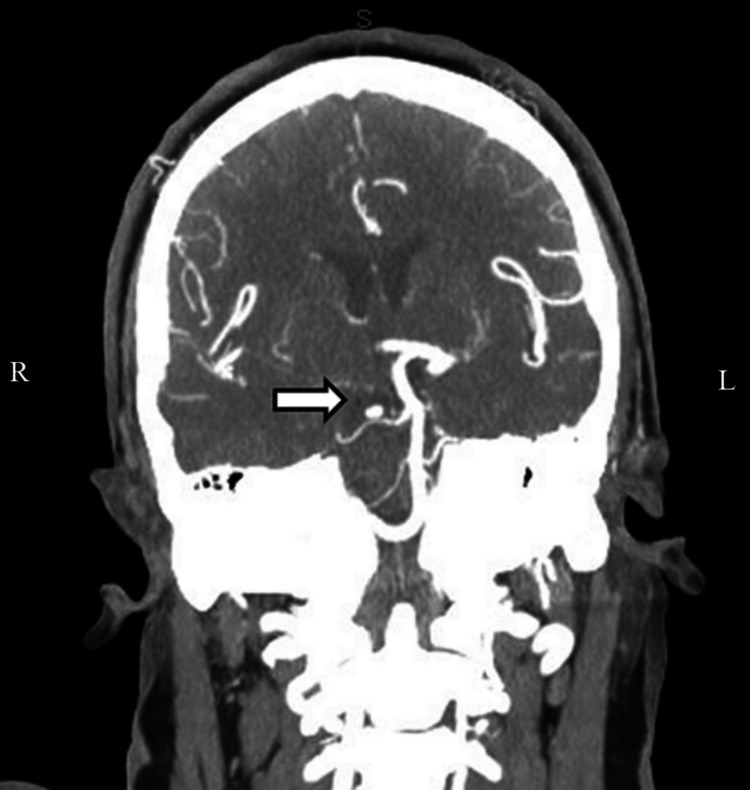
CTA head and neck with contrast - coronal section showing an absence of the right posterior cerebral artery. CTA: computed tomographic angiography.

**Figure 2:**
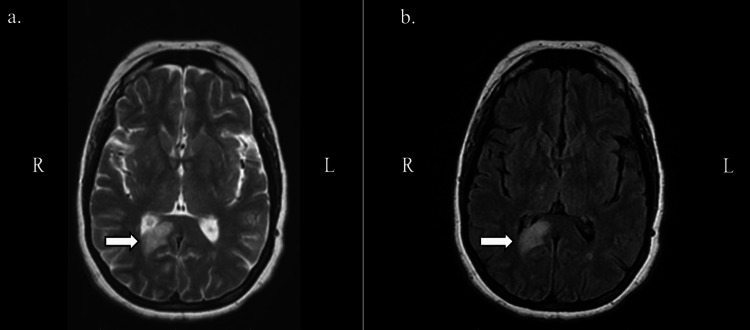
MR brain with and without contrast: inferior view. Transverse inferior view of MR brain: (a) fast spin echo and (b) FLAIR showing increased T2/FLAIR signal in the periventricular and subcortical white matter. MR: magnetic resonance, FLAIR: fluid-attenuated inversion recovery.

**Figure 3:**
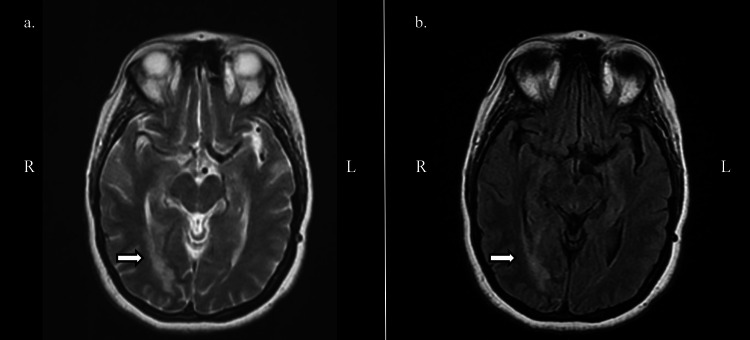
MR brain with and without contrast: superior view. Transverse superior view of MR brain (a) FSE and (b) FLAIR showing increased T2/FLAIR signal in the periventricular and subcortical white matter. MR: magnetic resonance, FLAIR: fluid-attenuated inversion recovery, FSE: fast spin echo.

**Figure 4:**
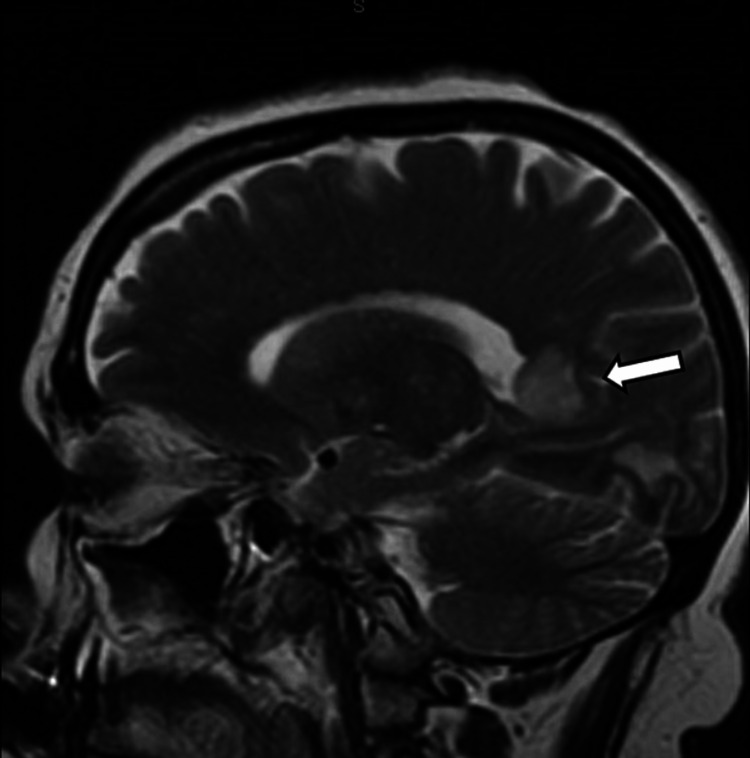
MR brain with contrast. Sagittal view of MR brain showing increased T2/FLAIR signal in the periventricular and subcortical white matter. MR: magnetic resonance, FLAIR: fluid-attenuated inversion recovery.

The results of the original CT done on the day of the incident were unable to be obtained; thus, an initial CT head without contrast was ordered for this ED presentation. The CT showed interval development of decreased attenuation in the right occipital lobe. Due to the findings, a CTA of the head and neck with contrast was ordered, which revealed an absence of the right posterior cerebral artery (PCA) (Figure 1). Neurology was consulted and recommended an MR brain with and without contrast. The MR brain showed increased T2/fluid-attenuated inversion recovery (FLAIR) signal in the periventricular and subcortical white matter. The MR impression was noted as a right occipital lobe infarct with cortical enhancement most consistent with subacute chronicity, as well as white matter abnormality which was advanced for the patient's age (Figures 2, 3, 4). After obtaining the MR brain, neurology recommended an MRA head and neck with MRV with and without contrast.

**Figure 5:**
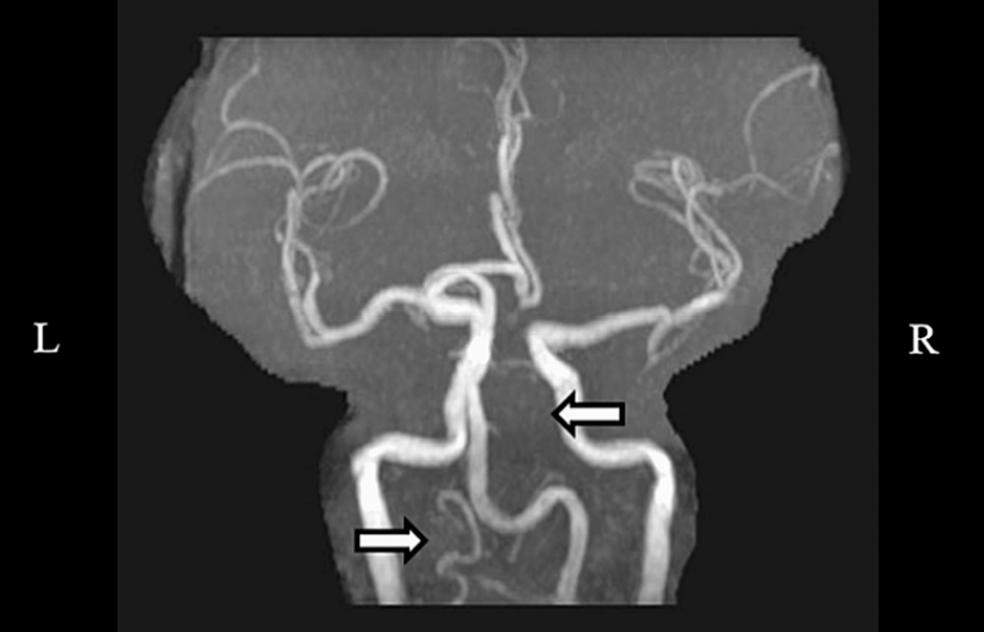
MRA head and neck MRA head and neck showing a developmentally slightly diminutive left vertebral artery and occluded right posterior cerebral artery, which was occluded at the P1-P2 junction. MRA: magnetic resonance angiography.

The MRA head and neck showed a developmentally slightly diminutive left vertebral artery that functionally terminates in the left posterior inferior cerebellar artery. Additionally, the fetal origin of the right PCA was found to be occluded at the P1-P2 junction. The MRA impression was a fetal origin right PCA, which was occluded at the P1-P2 junction, left vertebral artery developmental variant, and partially visualized subacute infarction of the right occipital lobe (Figure 5). Subsequently, the patient was admitted to the hospital for further evaluation and treatment.

During her hospital stay, the patient was started on aspirin 81 mg, atorvastatin 80 mg, and clopidogrel 75 mg. In addition, she continued her home medications of metoprolol 200 mg, nifedipine 90 mg, and spironolactone 25 mg. After a thorough exam and review of diagnostic findings, the neurologist concluded that the patient was most likely suffering from a subacute stroke secondary to the episode of IPV. The patient likely had a small right PCA that closed off due to the trauma. The patient was discharged home and continued all medications. She was also scheduled to receive a hypercoagulable workup, transesophageal echocardiogram with a bubble study to rule out patent foramen ovale and other atrial abnormalities, and an MRI with and without contrast in two to three months.

## Discussion

The 2016/2017 Report on IPV from the National Intimate Partner and Sexual Violence Survey found that nearly half of women (47.3%) and more than two-fifths of men (44.2%) in the United States had experienced IPV in their lifetime. Additionally, over two-fifths of women (42.0%) and men (42.3%) reported experiencing physical IPV in their lifetime. Severe acts of physical IPV include being hit with a fist or other hard object, kicked, slammed against things, beaten, choked/suffocated, or having a knife or gun used on them. Of the 42.0% of women who have experienced physical IPV, almost one in three (32.5% or 40.5 million) experienced severe physical violence, with 18.9% reporting being hit with a fist or something hard. In comparison, 39% of men (46.1 million) who experienced physical IPV also experienced severe physical violence, with 16.9% reporting being hit with a fist or something hard [2].

Health consequences from IPV include a variety of psychological and non-psychological conditions. While the link between IPV and psychological conditions such as PTSD, depression, and suicidal ideations has been established in numerous studies, the association between IPV and neurological complications is less clear [6-9]. Dillon et al. reported that women who are victims of IPV are more likely to report chronic headaches in their lifetime [9]. In addition, a large retrospective review of de-identified electronic health records across the United States conducted by Karakurt et al. found that victims of IPV were more likely to report headaches, migraines, and other neurological symptoms [10].

These findings, however, are not consistent across the literature. A study by Campbell et al. examined the association between IPV and possible traumatic brain injury (TBI) on different central nervous system symptoms. The study included 901 women, of which 543 were cases who had experienced IPV in their lifetime. Among these cases, 31% (n = 168) had experienced head trauma. However, the study found no significant difference in headache prevalence between the cases and controls (p=0.416) or between those with probable or no probable TBI (p = 0.108) [11]. A similar study was conducted by Gerber et al. in 2012, with participants recruited from a women's headache clinic. Their findings indicated that IPV was not directly associated with headache severity. However, patients who had recently experienced IPV scored 1.6 points higher on the Migraine Disability Assessment (MIDAS) measure of headache severity than those who had not recently experienced IPV. Additionally, patients who had experienced any IPV in their lifetime scored 9 points higher on the MIDAS. Gerber et al. concluded that although these results were not statistically significant, the headaches had a significant impact on the patients' lives [12].

Other reported and studied neurological complications of IPV include recurrent dizziness, blacking out, memory loss, difficulties concentrating, hearing problems, and vision problems [11,13-15]. A study conducted by Al-Modallal in 2016 focused on the health effects of IPV on Palestinian Refugees and found that women who experienced physical, sexual, and psychological IPV had a significantly higher rate of recurrent dizziness compared to women who did not experience IPV [13]. These findings align with other studies, such as Campbell et al., who reported that 60% of women who experienced IPV had episodes of dizziness, compared to 37% of those who did not experience IPV [11]. The uniqueness of Al-Modallal's findings lies in the observation that the highest rates of recurrent dizziness were found in women who experienced psychological IPV (75%). This suggests that certain neurological symptoms may not necessarily require physical violence and could instead be a manifestation of all types of IPV [13].

Similarly, in line with the presented case, Campbell et al. found that female victims of IPV had significantly higher rates of vision problems compared to those who did not experience IPV. According to their study, 49% of women who experienced IPV and 57% of women who experienced IPV with probable TBI reported episodes of vision problems, in contrast to 33% and 41% of women who did not experience IPV [11]. Given the mechanism of injury in the presented case, the patient would be classified as having a probable TBI and, therefore, was at increased risk of experiencing dizziness, vision changes, and, according to the literature, headaches. Additionally, the patient also suffered from an ischemic stroke, most likely as a consequence of her experienced IPV. It is noteworthy that the connection between IPV and ischemic stroke has not been previously reported in adults. Nevertheless, cases of ischemic stroke following TBI have been documented.

Two reported studies have analyzed the risk of ischemic stroke in patients who had experienced TBI. Kowalski et al. conducted a study in 2017 on a cohort of 6488 patients who had suffered from TBI and found that 159 (2.5%) of these patients were diagnosed with acute ischemic stroke before hospital discharge [16]. Notably, the identification of stroke occurred at a median of 25 days after the injury, indicating that symptom presentation and proper identification might be delayed [16]. In the presented case, it remains uncertain whether the patient's stroke would have been detected during her initial hospital visit or if the complete occlusion of the PCA occurred over time.

A similar study conducted by Burke et al. in 2013 demonstrated a more direct link between TBI and ischemic stroke. In their study of 1,173,353 trauma subjects, 436,630 (37%) had experienced TBI. The findings revealed that TBI had a significant hazard ratio of 1.31 for ischemic stroke compared to the non-TBI group [17]. These results were consistent with a Taiwanese study in 2011, which reported that TBI was independently associated with a 10.21 times greater risk of stroke at three months compared to those without a history of TBI [18].

While the reported literature does analyze TBI and stroke, TBI is primarily secondary to motor vehicle accidents, falls, sports, or flying objects [16-18]. None of the reviewed studies conducted subgroup analysis on TBI secondary to IPV or other forms of violence. While not reported in adult literature, abusive head trauma has been linked to stroke in the pediatric population [19,20]. A study by Khan et al. in 2017 found that 28% of pediatric patients who experienced abusive head trauma later suffered from one or more strokes [20].

## Conclusions

Our case is the first of its kind reported in the current literature review and establishes connections with earlier studies on abuse, head injury, and neurological symptoms. Victims of IPV have been shown to experience increased rates of various neurological symptoms, including dizziness, headaches, and vision changes, all of which our patient experienced. Additionally, while not directly linked to IPV, TBI has been associated with an increased risk of ischemic stroke in the adult population, and abusive head trauma has been linked to stroke in the pediatric population. Although this suggests that traumatic head injuries secondary to IPV and abuse may elevate the likelihood of stroke, further studies will be necessary to confirm this association. In summary, IPV is an escalating public health concern, and victims of IPV may encounter lifelong neurological effects stemming from physical, sexual, and psychological violence.

## References

[1] Breiding MJ, Basile KC, Smith SG, Black MC, Mahendra RR (2015). Intimate Partner Violence Surveillance: Uniform Definitions and Recommended Data Elements, Version 2.0. Centers for Disease Control and Prevention National Center for Injury Prevention and Control.

[2] Leemis RW, Friar N, Khatiwada S (2022). The National Intimate Partner and Sexual Violence Survey: 2016/2017 Report on Intimate Partner Violence. National Center for Injury Prevention and Control Centers for Disease Control and Prevention.

[3] Potter LC, Morris R, Hegarty K, García-Moreno C, Feder G (2021). Categories and health impacts of intimate partner violence in the World Health Organization multi-country study on women's health and domestic violence. Int J Epidemiol.

[4] (2023). Violence against women. https://www.who.int/news-room/fact-sheets/detail/violence-against-women.

[5] Ellsberg M, Jansen HA, Heise L, Watts CH, Garcia-Moreno C; WHO Multi-country Study on Women's Health and Domestic Violence against Women Study Team (2008). Intimate partner violence and women’s physical and mental health in the WHO multi-country study on women’s health and domestic violence: an observational study. Lancet.

[6] Bacchus LJ, Ranganathan M, Watts C, Devries K (2018). Recent intimate partner violence against women and health: a systematic review and meta-analysis of cohort studies. BMJ Open.

[7] Iverson KM, Dardis CM, Pogoda TK (2017). Traumatic brain injury and PTSD symptoms as a consequence of intimate partner violence. Compr Psychiatry.

[8] Lutwak N (2018). The psychology of health and illness: the mental health and physiological effects of intimate partner violence on women. J Psychol.

[9] Dillon G, Hussain R, Loxton D, Rahman S (2013). Mental and physical health and intimate partner violence against women: a review of the literature. Int J Family Med.

[10] Karakurt G, Patel V, Whiting K, Koyutürk M (2017). Mining electronic health records data: domestic violence and adverse health effects. J Fam Violence.

[11] Campbell JC, Anderson JC, McFadgion A (2018). The effects of intimate partner violence and probable traumatic brain injury on central nervous system symptoms. J Womens Health (Larchmt).

[12] Gerber MR, Fried LE, Pineles SL, Shipherd JC, Bernstein CA (2012). Posttraumatic stress disorder and intimate partner violence in a women's headache center. Women Health.

[13] Al-Modallal H (2016). Effect of intimate partner violence on health of women of Palestinian origin. Int Nurs Rev.

[14] Stubbs A, Szoeke C (2022). The effect of intimate partner violence on the physical health and health-related behaviors of women: a systematic review of the literature. Trauma Violence Abuse.

[15] Sugg N (2015). Intimate partner violence: prevalence, health consequences, and intervention. Med Clin North Am.

[16] Kowalski RG, Haarbauer-Krupa JK, Bell JM (2017). Acute ischemic stroke after moderate to severe traumatic brain injury: incidence and impact on outcome. Stroke.

[17] Burke JF, Stulc JL, Skolarus LE, Sears ED, Zahuranec DB, Morgenstern LB (2013). Traumatic brain injury may be an independent risk factor for stroke. Neurology.

[18] Chen YH, Kang JH, Lin HC (2011). Patients with traumatic brain injury: population-based study suggests increased risk of stroke. Stroke.

[19] Balachandran A, Kalyanshettar S, Patil S, Shegji V (2016). Ischemic stroke in confederation with trivial head trauma. Case Rep Pediatr.

[20] Khan NR, Fraser BD, Nguyen V, Moore K, Boop S, Vaughn BN, Klimo P Jr (2017). Pediatric abusive head trauma and stroke. J Neurosurg Pediatr.

